# Hormone replacement therapy and risks of oesophageal and gastric adenocarcinomas

**DOI:** 10.1038/sj.bjc.6602906

**Published:** 2005-12-13

**Authors:** M Lindblad, L A García Rodríguez, E Chandanos, J Lagergren

**Affiliations:** 1 Unit of Oesophageal and Gastric Research, Department of Molecular Medicine and Surgery, Karolinska Institutet, Stockholm, Sweden; 2 Centro Español de Investigación Farmacoepidemiológica, Madrid, Spain; 3 Department of Medical Epidemiology and Biostatistics, Karolinska Institutet, Stockholm, Sweden

**Keywords:** oestrogen, hormone replacement therapy, gastric cancer, oesophageal adenocarcinoma

## Abstract

Oesophageal and gastric adenocarcinoma share an unexplained male predominance, which would be explained by the hypothesis that oestrogens are protective in this respect. We carried out a nested case–control study of hormone replacement therapy (HRT) among 299 women with oesophageal cancer, 313 with gastric cancer, and 3191 randomly selected control women, frequency matched by age and calendar year in the General Practitioners Research Database in the United Kingdom. Data were adjusted for age, calendar year, tobacco smoking, alcohol consumption, body mass index, hysterectomy, and upper gastrointestinal disorders. Among 1 619 563 person-years of follow-up, more than 50% reduced risk of gastric adenocarcinoma was found among users of HRT compared to nonusers (odds ratio (OR), 0.48, 95% confidence interval (CI) 0.29–0.79). This inverse association appeared to be stronger for gastric noncardia (OR 0.34, 95% CI 0.14–0.78) and weaker for gastric cardia tumours (OR 0.68, 95% CI 0.23–2.01). There was no association between HRT and oesophageal adenocarcinoma (OR 1.17, 95% CI 0.41–3.32).

The male predominance is a striking and unexplained feature of oesophageal and gastric adenocarcinomas, the sex ratio being 6 : 1 in both oesophageal and gastric cardia adenocarcinoma, and 2 : 1 in gastric noncardia adenocarcinoma (Corley and Buffler, 2001; Parkin, 2001). The 3 : 1 male predominance in oesophageal *squamous-cell* carcinoma is explicable by sex differences in the prevalence of the main risk factors, that is, use of tobacco and alcohol (Muir and McKinney, 1992; Gammon *et al*, 1997; Lagergren *et al*, 2000). However, no corresponding sex difference in the established risk factors for oesophageal or gastric adenocarcinoma can explain their male predominance (EUROGAST, 1993; Moller *et al*, 1994; Lagergren *et al*, 1999a, 1999b; Kelley and Duggan, 2003; Crew and Neugut, 2004). It has been hypothesised that female sex hormones, mainly oestrogens, may explain or contribute to these sex differences. In two prostate cancer cohort studies, of men heavily exposed to oestrogens, there was no reduction in the risk of oesophageal adenocarcinoma (Lagergren and Nyren, 1998), although a decreased risk of gastric adenocarcinoma has been reported (Lindblad *et al*, 2004). An increased male-to-female ratio in gastric adenocarcinoma has been claimed as corresponding to a 10- to 15-year delay among females compared with males, possibly due to a protective effect of female sex hormones (Sipponen and Correa, 2002). Studies on menstrual and reproductive factors have suggested associations with the risk of gastric adenocarcinoma (Miller *et al*, 1980; Plesko *et al*, 1985; La Vecchia *et al*, 1994; Palli *et al*, 1994; Heuch and Kvale, 2000; Inoue *et al*, 2002; Kaneko *et al*, 2003). Based on the hypothesis that sex hormones may be involved in the aetiology of oesophageal or gastric adenocarcinoma, we investigated whether hormone replacement therapy (HRT) protects women against either of these tumours.

## MATERIALS AND METHODS

### General practice research database

We collected our data from the General Practice Research Database (GPRD) in the United Kingdom (UK). This database has been described in detail elsewhere (Garcia Rodriguez and Perez Gutthann, 1998). The large number of person-years of follow-up makes the GPRD a valuable resource even for the study of rare diseases, and we have previously used this database to study risk factors for oesophageal and gastric cancer (Lindblad *et al*, 2005). The computerised information in the GPRD includes prospective recordings of diagnoses, prescriptions of drugs, demographics, details of every general practitioner's (GPs) consultation, results from laboratory tests, hospital letters, and a free text section. Diagnoses are coded by a modification of the Oxford Medical Information System (OXMIS) and the Read classification systems. A drug dictionary based on data from the Prescription Pricing Authority is used to code drugs. All prescriptions are automatically entered into the GPRD since they are directly generated from the participating GP's computer, thus ensuring a complete recording. Validation studies have shown that the GPRD data are of high quality (Jick *et al*, 2003). More than 90% of all referrals are entered into the GPs computer with the specialist's diagnosis carefully coded (Jick *et al*, 1991).

### Study cohort

During the study period, January 1994 through December 2001, we identified all women in the GPRD aged between 50 and 84 years. To be included in the study cohort, the person had to have been enrolled with the GP for at least 2 years, and with at least 1 year of computerised prescription history. Any person with a cancer history recorded in the GPRD before start date was excluded. All women in the study cohort were followed up until any of the following events occurred: (1) detection of an oesophageal or gastric cancer, (2) detection of any other cancer, (3) age of 85 years, (4) death, or (5) end of study period (31 December 2001), whichever occurred first.

### Identification of study participants

Within the study period, a computerised search identified 705 female subjects with a diagnosis code of oesophageal or gastric cancer. To verify and further classify the tumours, all computerised patient profiles of these patients were manually reviewed by one investigator (ML) who was kept blinded to the exposure data. Moreover, GPs sent additional information regarding case patients identified by the computer (*N*=346). All available information about the tumour site and histology, as well as paper-based information (e.g. operation and pathology reports and letters from specialists) were reviewed. Patients were excluded if: (1) the tumour was benign, (2) the origin of a cancer was unknown, (3) the tumour was a metastasis, (4) the patient had another, concurrent cancer, (5) the cancer was diagnosed before the start date, or (6) the histological type was not adenocarcinoma or squamous-cell carcinoma. The index date among our case patients was set to be the date when the tumour was first recorded or when the manual review revealed an earlier date of diagnosis. All persons received a random date within the study period, and if it was within that individual's eligible person-time, she was marked as an eligible control subject. Thereafter, we randomly selected control subjects and their random dates were set as index dates. The control participants were frequency matched on age (within 1 year) and same calendar year.

### Drug exposure definition

Only drug exposure preceding the index date was considered in the study. The information recorded in the GPRD, drug medications and HRT included, could date back to the late 1980s when the database was initiated. HRT included oral oestrogens, transdermal oestradiol, oestradiol implants, and tibolone, and use was classified as nonuse or ever use when there was no or any recorded use in the database, respectively. Ever users were further categorised as current users if use of HRT had been within the year prior to index date, and past users if the most recent use was before that. Duration of HRT use was calculated summing the periods of consecutive prescriptions among ever users, grouped into two levels: less than 3 years of treatment duration and 3 years and more.

### Statistical analyses

Unconditional logistic regression was conducted to calculate odds ratios (ORs) with 95% confidence intervals (CIs). In multivariable analyses, all estimates of risk were adjusted for the following potential confounding factors: age (in 10-year intervals), calendar year, tobacco smoking (categorised in four groups: nonsmoker, current smoker, ex-smoker, or unknown), alcohol consumption (categorised in five groups: 0–2, 3–15, 16–34, >34 U day^−1^, or unknown, where one unit corresponded to 10 ml or 7.9 g of pure ethanol), body mass index (BMI) (categorised in five groups: <20, 20–24.9, 25–29.9, >30 kg m^−2^, or unknown), hysterectomy (categorised in two groups: yes or no), and upper gastrointestinal disorders (categorised in two groups: never or ever). Upper gastrointestinal disorders were defined as any recording in the GPRD of gastrooesophageal reflux disease, peptic ulcer, dyspepsia, or prescription of acid-suppressing drugs (proton-pump inhibitors or H_2_-receptor blockers).

### Ethics

The Scientific and Ethical Advisory Group (SEAG) in the UK approved the Study.

## RESULTS

### Study participants

Among 1 619 563 person-years of follow-up, the computerised search identified 705 women with oesophageal or gastric cancer. After excluding 93 during the manual review for any of the previously stated reasons, 299 were classified as oesophageal cancer and 313 as gastric cancer. The crude incidence rate was 18.5/100 000 person-years for oesophageal cancer and 19.3/100 000 person-years for gastric cancer. Oesophageal tumours were further classified by histological type, and gastric tumours into gastric subsite as presented in Table 1. Some basic characteristics of the study participants are shown in Table 1. The median ages among patients with oesophageal cancer, gastric cancer, and control subjects were 74, 73, and 74 years, respectively, and no striking differences were noted between the cancer subgroups. In the youngest age group (50–59 years), gastric cardia were over-represented and oesophageal adenocarcinomas were under-represented. Never smokers were more frequent among control participants than among all cancer subgroups. The prevalence of obesity (BMI>30 kg m^−2^) and of upper gastrointestinal disorders was higher in oesophageal and gastric cardia adenocarcinomas than among controls, oesophageal squamous-cell carcinomas, and gastric noncardia adenocarcinomas. The median time of data collection between first drug prescription and index date was similar between cases (2323 days) and controls (2272 days).

### HRT and oesophageal cancer

HRT was not associated with the risk of oesophageal cancer of any histological type (OR 0.84, 95% CI 0.51–1.38) as shown in Table 2. Multivariable analysis revealed an OR of 1.17 (95% CI 0.41–3.32) for oesophageal adenocarcinoma among ever users of HRT compared to nonusers. The corresponding OR for oesophageal squamous-cell carcinoma was 0.93 (95% CI 0.40–2.16). Adjustment for all covariates listed in the Materials and Methods section did not materially change the age-adjusted risk estimates, indicating lack of strong confounding by these covariates (Table 2). The limited numbers of exposed cases and controls prohibited valid analyses of duration and recency of HRT use, but the point estimates were constant over treatment duration, while lower estimates were found among current users (OR 0.68).

### HRT and gastric cancer

Ever use of HRT was associated with a reduced risk of gastric adenocarcinoma by 52% compared to nonusers (OR 0.48, 95% CI 0.29–0.79) (Table 3). The corresponding analysis restricted to gastric cardia adenocarcinoma indicated a 32% reduced risk without statistical significance (OR 0.68, 95% CI 0.23–2.01). The 66% reduced risk of gastric noncardia adenocarcinoma was statistically significantly (OR 0.34, 95% CI 0.14–0.78). Stratified analysis by age groups and tobacco smoking status revealed a more pronounced inverse association in older compared to younger women, while no difference in risk was found between smokers and nonsmokers (Table 4). For HRT use of less than 3 years, OR was 0.34 (95% CI 0.17–0.67) of gastric cancer, while for at least 3 years it was 0.72 (95% CI 0.38–1.38). Current users of HRT were associated with a reduced risk of gastric cancer (OR 0.56, 95% CI 0.33–0.96) as well as past users (OR 0.25, 95% CI 0.09–0.70).

## DISCUSSION

This study indicates that use of HRT was associated with a markedly reduced risk of gastric adenocarcinoma, possibly greater in gastric tumours with a noncardia location, while no association was observed for oesophageal adenocarcinoma.

The low exposure prevalence of HRT and the low incidence of oesophageal and gastric adenocarcinoma in females might explain the lack of previous studies of this topic. Our large sample size was necessary to provide informative results. The reported incidence of adenocarcinoma of the oesophagus and gastric cardia in the UK is higher than that of any other country worldwide (Berrino *et al*, 1999). Still, the limited number of exposed affected women in our study prohibited extensive subgroup analyses, including the influence of duration of HRT or HRT regimen. The prospective exposure assessment, that is, before cancer occurrence, is the main strength of the study. Also, our manual review of all case patients should reduce misclassification of cancer, although a number of missing values remained on specific histology and subsite. The risk of tumour misclassification was highest for gastric cardia adenocarcinoma, so our estimates for this site are more prone to such error. The lack of a strong inverse association between HRT and oesophageal cancers in our study provides some reassurance that selection bias does not explain the inverse association with gastric cancer. Any remaining tumour misclassification should not be associated with HRT use and could therefore, at the most, dilute our results toward null. Several potential confounding factors were considered, but not *Helicobacter pylori* infection or socioeconomic status. *H pylori* infection, an established risk factor for gastric noncardia adenocarcinoma (Uemura *et al*, 2001), has also been reported to be associated with a reduced risk of oesophageal adenocarcinoma, but not of gastric cardia adenocarcinoma (Hansson *et al*, 1993; Ye *et al*, 2004). *H pylori* infection is positively associated with low social class (Boffetta, 1997), which in turn could be associated with reduced HRT use. As we could not adjust for these factors, our results could be distorted by residual confounding. However, our lack of association between HRT and oesophageal squamous-cell carcinoma, well known to be associated with low social class, does not support a major role for confounding by social class.

Few studies of hormonal factors and risk of oesophageal adenocarcinoma have been published. Our lack of a clear effect of HRT on the risk of oesophageal cancer is in contrast to a study which found that use of HRT, oral contraceptives, and age at menopause were all inversely related with the risk of oesophageal squamous-cell carcinoma (Gallus *et al*, 2001). The small sample size, the lack of data regarding oesophageal adenocarcinoma, and the retrospective exposure assessment in this study might explain the differences from our study. In line with our current findings is the lack of association between oestrogen treatment and oesophageal adenocarcinoma in our cohort study of prostate cancer patients (Lagergren and Nyren, 1998). There have been somewhat more studies of hormonal influences on gastric cancer. Our findings accord with the age-group-specific male-to-female ratio unique for gastric adenocarcinomas (Sipponen and Correa, 2002). In line with our findings is the decreased risk of gastric adenocarcinoma in our cohort of oestrogen-exposed prostate cancer patients (Lindblad *et al*, 2004) and a protective trend by HRT on gastric cancer (La Vecchia *et al*, 1994; Fernandez *et al*, 2003). However, results from studies of menstrual, reproductive factors, and parity and risk of gastric cancer are partly conflicting (Miller *et al*, 1980; Plesko *et al*, 1985; La Vecchia *et al*, 1994; Palli *et al*, 1994; Heuch and Kvale, 2000; Inoue *et al*, 2002; Kaneko *et al*, 2003). A longer period of fertility among females, that is, the time from menarche to menopause, increases the lifetime exposure to endogenous oestrogens and has been suggestive of a reduced gastric cancer risk (La Vecchia *et al*, 1994; Palli *et al*, 1994; Inoue *et al*, 2002; Kaneko *et al*, 2003), although a lack of such association, and an inverse association with age at menarche has also been reported (Heuch and Kvale, 2000). Multiparity has been associated with both an increased risk of gastric cancer in several studies (Plesko *et al*, 1985; La Vecchia *et al*, 1994; Palli *et al*, 1994; Heuch and Kvale, 2000; Inoue *et al*, 2002), and a reduced risk of gastric cancer (Kaneko *et al*, 2003). Experimental data in rats (Furukawa *et al*, 1982; Campbell-Thompson *et al*, 1999) suggest that oestrogen may prevent gastric cancer development.

The inverse association of HRT was particularly strong for gastric noncardia adenocarcinoma. It is not surprising that the associations between HRT and subsites of adenocarcinoma of the oesophagus, gastric cardia, and gastric noncardia differ. Marked differences in risk factor profiles exist between these tumour sites (Fuchs and Mayer, 1995; Enzinger and Mayer, 2003). The evidence of hormonal influence is strongest for gastric noncardia adenocarcinoma. The seemingly stronger protective effect of HRT among older women, who are more likely to have longer exposures, might suggest that a longer dose–response relation exists. This, however, could not be confirmed though our numbers were limited.

The mechanism of a potential protective effect of HRT on gastric cancer is unknown. Oestrogen receptors have been identified in normal and cancer tissue of the oesophagus and stomach (Korenaga *et al*, 1998; Akgun *et al*, 2002; Matsuyama *et al*, 2002). Oestrogens may prevent colon cancer by decreasing bile acid concentration or by direct effects on the colonic mucosa, as suggested by *in vitro* studies (McMichael and Potter, 1980; Nanda *et al*, 1999), and this may also apply in gastric adenocarcinoma. The fact that the protective effect of HRT is stronger for noncardia tumours, that is, those closer to the bile duct, might support this possibility. Women have a lower gastric acid secretion than men, and oestrogen reduces acid gastric secretion and the mass of parietal cells in rats (Adeniyi, 1991), so oestrogen may affect gastric acid production.

The relation between HRT and gastric adenocarcinoma warrants further research before any clinical implications would be appropriate, particularly given the initial enthusiasm about the seemingly protective effect of HRT against cardiovascular disease, which was later not supported (Nelson *et al*, 2002).

In conclusion, our large study using a prospective database indicates that HRT is associated with a risk reduction of gastric adenocarcinoma, particularly of the noncardia site, while no association was found for oesophageal adenocarcinoma. As we could not control for *H. pylori* infection or social class, our findings must be regarded as tentative. If oestrogens truly and strongly protect against the development of gastric adenocarcinoma, its male predominance may be partly explained by the endogenous oestrogen exposure during women's fertile years. Further research on the potential benefits and harm of sex hormones in relation to gastric cancer may be warranted.

## Figures and Tables

**Table 1:** Selected basic characteristics among case patients and control participants

		**Oesophageal cancer**				**Gastric adenocarcinoma**			
	**Controls**	**Numbers (%)**				**Numbers (%)**			
**Characteristic**	**Numbers (%)**	**Adenocarcinoma**	**Squamous-cell carcinoma**	**Unknown histology**	**Total**	**Cardia**	**Noncardia**	**Unknown subsite**	**Total**
Total	3191 (100)	58 (100)	74 (100)	167 (100)	299 (100)	43 (100)	116 (100)	154 (100)	313 (100)
*Age (years)*									
Median	74 years	75 years	72 years	75 years	74 years	72 years	72 years	75 years	73 years
50–59	359 (11.2)	2 (3.4)	7 (9.5)	15 (9.0)	24 (8.0)	6 (13.9)	19 (16.4)	18 (11.7)	43 (13.7)
60–69	714 (22.4)	12 (20.7)	22 (29.7)	31 (18.6)	65 (21.8)	12 (27.9)	30 (25.8)	27 (17.5)	69 (22.1)
70–79	1362 (42.7)	32 (55.2)	34 (46.0)	74 (44.3)	140 (46.8)	18 (41.9)	40 (34.5)	65 (42.2)	123 (39.3)
80–84	756 (23.7)	12 (20.7)	11 (14.8)	47 (28.1)	70 (23.4)	7 (16.3)	27 (23.3)	44 (28.6)	78 (24.9)
									
*Tobacco smoking status*									
Never	1942 (60.9)	31 (53.4)	37 (50.0)	75 (44.9)	143 (47.8)	19 (44.2)	57 (49.1)	81 (52.6)	157 (50.1)
Ex-smoker	190 (6.0)	7 (12.1)	4 (5.4)	11 (6.6)	22 (7.4)	7 (16.3)	12 (10.3)	17 (11.0)	36 (11.5)
Smoker	400 (12.5)	11 (19.0)	14 (18.9)	36 (21.6)	61 (20.4)	9 (20.9)	28 (24.2)	24 (15.6)	61 (19.5)
Unknown	659 (20.6)	9 (15.5)	19 (25.7)	45 (26.9)	73 (24.4)	8 (18.6)	19 (16.4)	32 (20.8)	59 (18.9)
									
*Alcohol consumption (U day*^−*1*^ a)									
0–2	1758 (55.1)	31 (53.4)	33 (44.6)	84 (50.3)	148 (49.5)	19 (44.2)	70 (60.3)	90 (58.5)	179 (57.2)
3–15	395 (12.4)	14 (24.1)	10 (13.5)	13 (7.8)	37 (12.4)	7 (16.3)	16 (13.8)	15 (9.7)	38 (12.1)
16–34	33 (1.0)	2 (3.5)	1 (1.4)	1 (0.6)	4 (1.3)	2 (4.6)	1 (0.9)	2 (1.3)	5 (1.6)
35+	1 (0.0)	0 (0.0)	0 (0.0)	1 (0.6)	1 (0.3)	0 (0.0)	0 (0.0)	0 (0.0)	0 (0.0)
Unknown	1004 (31.5)	11 (19.0)	30 (40.5)	68 (40.7)	109 (36.5)	15 (34.9)	29 (25.0)	47 (30.5)	91 (29.1)
									
*Body mass index (kg m*^−*2*^)									
Less than 20	142 (4.5)	4 (6.9)	5 (6.8)	13 (7.8)	22 (7.4)	1 (2.3)	12 (10.3)	8 (5.2)	21 (6.7)
20–24.9	812 (25.4)	10 (17.2)	20 (27.0)	31 (18.5)	61 (20.4)	12 (27.9)	35 (30.2)	46 (29.9)	93 (29.7)
25–29.9	765 (24.0)	16 (27.6)	16 (21.6)	26 (15.6)	58 (19.4)	14 (32.6)	24 (20.7)	35 (22.7)	73 (23.3)
30+	396 (12.4)	13 (22.4)	2 (2.7)	15 (9.0)	30 (10.0)	7 (16.3)	11 (9.5)	17 (11.0)	35 (11.2)
Unknown	1076 (33.7)	15 (25.9)	31 (41.9)	82 (49.1)	128 (42.8)	9 (20.9)	34 (29.3)	48 (31.2)	91 (29.1)
									
*Hysterectomy*									
No	2735 (85.7)	52 (89.7)	67 (90.5)	153 (91.6)	272 (91.0)	38 (88.4)	107 (92.2)	133 (86.4)	278 (88.8)
Yes	456 (14.3)	6 (10.3)	7 (9.5)	14 (8.4)	27 (9.0)	5 (11.6)	9 (7.8)	21 (13.6)	35 (11.2)
									
*Upper gastrointestinal disorders* b									
Never	2056 (64.4)	25 (43.1)	41 (55.4)	99 (59.3)	165 (55.2)	16 (37.2)	59 (50.9)	63 (40.9)	138 (44.1)
Ever	1135 (35.6)	33 (56.9)	33 (44.6)	68 (40.7)	134 (44.8)	27 (62.8)	57 (49.1)	91 (59.1)	175 (55.9)

a One unit of alcohol corresponds to 10 ml or 7.9 g of pure ethanol.

b Upper gastrointestinal disorders were defined as any recording in the GPRD of gastrooesophageal reflux disease, peptic ulcer, dyspepsia, or prescription of acid-suppressing drugs (proton-pump inhibitors or H_2_-receptor blockers).

**Table 2:** Odds ratios (ORs) with 95% confidence intervals (CIs) on the association between use of hormone replacement therapy (HRT) and risk of oesophageal cancer

	**Control subjects**	**Oesophageal adenocarcinoma**		**Oesophageal squamous-cell carcinoma**		**Oesophageal cancer of unknown histology**		**Total oesophageal cancer**	
**Exposure**	***N* (%)**	***N* (%)**	**OR (95% CI)**	***N* (%)**	**OR (95% CI)**	***N* (%)**	**OR (95% CI)**	***N* (%)**	**OR (95% CI)**
*HRT*									
Never	2846 (89.2)	53 (91.4)	1.00 (reference)	66 (89.2)	1.00 (reference)	157 (94.0)	1.00 (reference)	276 (92.3)	1.00 (reference)
Ever	345 (10.8)	5 (8.6)	1.27 (0.46–3.48)^a^	8 (10.8)	0.94 (0.42–2.15)^a^	10 (6.0)	0.56 (0.28–1.15)^a^	23 (7.7)	0.78 (0.48–1.26)^a^
			1.17 (0.41–3.32)^b^		0.93 (0.40–2.16)^b^		0.65 (0.31–1.37)^b^		0.84 (0.51–1.38)^b^

a ORs adjusted only for age.

b ORs adjusted for age, calendar year, tobacco smoking, alcohol consumption, body mass index, hysterectomy, and upper gastrointestinal disorders.

**Table 3:** Odds ratios (ORs) with 95% confidence intervals (CIs) on the association between use of hormone replacement therapy (HRT) and risk of gastric adenocarcinoma

	**Control subjects**	**Gastric cardia adenocarcinoma**		**Gastric noncardia adenocarcinoma**		**Unknown subsite of gastric adenocarcinoma**		**Total gastric adenocarcinoma**	
**Exposure**	***N* (%)**	***N* (%)**	**OR (95% CI)**	***N* (%)**	**OR (95% CI)**	***N* (%)**	**OR (95% CI)**	***N* (%)**	**OR (95% CI)**
*HRT*									
Never	2846 (89.2)	38 (88.4)	1.00 (reference)	109 (94.0)	1.00 (reference)	143 (92.9)	1.00 (reference)	290 (92.6)	1.00 (reference)
Ever	345 (10.8)	5 (11.6)	0.85 (0.30–2.44)^a^		0.34 (0.15–0.77)^a^		0.61 (0.30–1.21)^a^		0.51 (0.32–0.83)^a^
			0.68 (0.23–2.01)^b^	7 (6.0)	0.34 (0.14–0.78)^b^	11 (7.1)	0.55 (0.27–1.12)^b^	23 (7.4)	0.48 (0.29–0.79)^b^

a ORs adjusted for age.

b ORs adjusted for age, calendar year, tobacco smoking, alcohol consumption, body mass index, hysterectomy, and upper gastrointestinal disorders.

**Table 4:** Odds ratios (ORs) with 95% confidence interval (CI) on the association between use of hormone replacement therapy and gastric adenocarcinoma stratified by age and tobacco smoking categories

	**Gastric adenocarcinoma**
**Stratified category**	**OR (95% CI)^a^**
*Age (years)*	
50–59	0.53 (0.25–1.13)
(Number of exposed cases/controls)	(16/173)
60+	0.38 (0.18–0.84)
(Number of exposed cases/controls)	(7/172)
	
*Tobacco smoking status*	*OR (95*% *CI)*^b^
Never	0.53 (0.26–1.08)
(Number of exposed cases/controls)	(11/214)
Ever	0.52 (0.24–1.12)
(Number of exposed cases/controls)	(13/107)

a Adjusted for calendar year, tobacco smoking, alcohol consumption, body mass index, hysterectomy, and upper gastrointestinal disorders.

b Adjusted for age, calendar year, alcohol consumption, body mass index, hysterectomy, and upper gastrointestinal disorders.
